# Évaluation en milieu rural au Congo de la fiabilité de la méthode de la goutte épaisse calibrée pour évaluer le niveau de la microfilarémie à *Loa loa*

**DOI:** 10.48327/mtsi.v3i1.2023.297

**Published:** 2023-03-23

**Authors:** Jérémy T. Campillo, Frédéric Louya, Paul Bikita, François Missamou, Michel Boussinesq, Sébastien D. S. Pion, Sébastien Bertout, Cédric B. Chesnais

**Affiliations:** 1 TransVIHMI, Université de Montpellier, Institut de recherche pour le développement (IRD), INSERM Unité 1175, 911 avenue Agropolis, 34000 Montpellier, France; 2 Programme national de lutte contre l'onchocercose, Direction de l’Épidémiologie et de la lutte contre la maladie, Ministère de la Santé et de la population, Brazzaville, République du Congo; 3 Laboratoire de Parasitologie et mycologie médicale, Université de Montpellier, Montpellier, France

**Keywords:** *Loa loa*, Microfilarémie, Microscopie, Fiabilité, Répétabilité, Reproductibilité, Sibiti, République du Congo, Afrique subsaharienne, L*oa loa*, Microfilaremia, Microscopy, Reliability, Repeatability, Reproducibility, Sibiti, Republic of the Congo, Sub-Saharan Africa

## Abstract

**Introduction-Justification:**

Le diagnostic de la microfilarémie à *Loa loa* consiste en l'observation, à l'aide d'un microscope, de microfilaires dans un échantillon de sang périphérique étalé sur une lame, puis coloré. Cette technique, appelée goutte épaisse, doit donner des résultats précis car ceux-ci guident la prise en charge clinique du patient.

**Matériel et méthode:**

Nous avons évalué sa fiabilité (reproductibilité et répétabilité) selon les critères du Comité français d'accréditation (COFRAC), en utilisant plusieurs séries de 10 lames positives à *L. loa*, choisies au hasard, et avons considéré les résultats au regard des exigences réglementaires.

**Résultats:**

Les coefficients de répétabilité estimés et acceptables (NB: plus la valeur est basse, meilleure est la répétabilité) étaient de 13,6% et 16,0%, respectivement. Les coefficients estimés et acceptables de fidélité intermédiaire (reproductibilité) étaient de 15,1% et 22,5%, respectivement. Le plus mauvais coefficient de fidélité intermédiaire était de 19,5% lorsque le paramètre de variation était le technicien effectuant les lectures (10,7% lorsque le critère de variation était la date de lecture). Le coefficient de variation inter-technicien parmi les 1876 lames positives à *L. loa* a été estimé à 13,2%. Le coefficient de variation inter-technicien considéré comme acceptable a été estimé à 18,6%.

**Discussion-Conclusion:**

Les coefficients de variabilité estimés étaient tous inférieurs aux coefficients acceptables calculés. Néanmoins, l'absence de références de laboratoire ne permet pas de conclure sur la qualité de ce diagnostic. Il est impératif de mettre en place une démarche qualité et une standardisation des procédures pour le diagnostic de la microfilarémie à *L. loa*, tant dans les pays endémiques que dans le reste du monde, où la demande de diagnostic augmente depuis des années.

## Abstract (en)

**Background-Rationale:**

The diagnosis of *Loa loa* microfilaremia consists in the observation, using a microscope, of microfilariae in a sample of peripheral blood spread on a slide and subsequently stained (the “blood smear technique”). The accurate quantification of *Loa loa* microfilaremia is important because the choice of the first intention treatment depends on the patient's microfilaremia: severe adverse events can occur in individuals with high microfilarial densities when treated with ivermectin or diethylcarbamazine, the latter drug being the only one which can definitively cure the infection. However, despite the widespread usage of this technique and its role in guiding clinical management of the patient, estimates of its reliability remain scarce.

**Materials and methods:**

We evaluated the reliability (reproducibility and repeatability) of blood smear technique using several sets of 10 *L. loo*-positive slides, randomly selected, and considered the results with regard to regulatory requirements. The slides had been prepared as part of a clinical trial conducted in Sibiti, Republic of Congo, a region where loiasis is endemic.

**Results:**

The estimated and acceptable coefficients of repeatability (NB: the lower, the better) were 13.6% and 16.0%, respectively. The estimated and acceptable coefficients of intermediate reliability (reproducibility) were 15.1% and 22.5%, respectively. The poorest coefficient of intermediate reliability was 19.5% when the tested parameter was related to the technician who performed the readings (10.7% when the reading day was changed). The inter-technician coefficient of variation assessed using 1876 *L. loo*-positive slides was 13.2%. The coefficient of inter-technician variation considered acceptable was estimated at 18.6%. Discussion-Conclusion. All estimated coefficients of variability were lower than the calculated acceptable coefficients suggesting reliability of the technique, although the lack of laboratory references precludes any conclusion on the quality of this diagnosis. It is imperative to implement a quality system and standardization of procedures for the diagnosis of *L. loo* microfilaremia, both in endemic countries and in the rest of the world, where the demand for diagnosis has been increasing for years.

## Introduction-Justification

L'aire de transmission de la loase, parasitose causée par la filaire *Loo loo*, est strictement limitée à l'Afrique centrale. Cependant, l'augmentation des mobilités humaines entre l'Afrique centrale et l'Europe implique l'augmentation des cas importés de loase en Europe [2, 15, 17, 24]. En 2018, la Haute autorité de santé française a publié un rapport mentionnant que l'examen microscopique direct d'un échantillon de sang pour rechercher les microfilaires (Mfs), stades larvaires du parasite, « semble utilisé en première intention pour les personnes provenant de zones endémiques (par exemple les migrants, les expatriés, les voyageurs, etc.), en cas de suspicion clinique et d'hyperéosinophilie associée » [18]. Cette technique est recommandée par la France [12], l'Australie [8] et le Royaume-Uni [9] pour la prise en charge des patients revenant de zones endémiques. L'examen peut se faire sur du sang frais, sur frottis mince ou goutte épaisse (GE) colorée, ou sur du sang traité par une technique de concentration. L'examen d'une GE, technique la plus fréquemment utilisée dans les pays endémiques pour diagnostiquer une infection à *L. loo*, n'a jamais été soumis à une évaluation réglementaire.

En 2015, l'Agence nationale de sécurité du médicament et des produits de santé (ANSM) a publié un rapport de contrôle de qualité sur les analyses de biologie médicale. Sur les 257 laboratoires ayant participé à l’étude, 83,7% ont été capables de diagnostiquer la présence de Mfs de *L. loo* par l'examen d'un frottis sanguin coloré au May-Grünwald-Giemsa, et 16,3% des laboratoires ont fourni des diagnostics erronés: autres espèces de filaires (*Onchocerca volvulus, Wuchererio boncrofti, Monsonello ozzordi*), *Plosmodium* spp. ou Mfs sans précision d'espèce [1]. Or, non seulement l'identification du parasite est primordiale pour le diagnostic de la microfilarémie à *L. loa*, mais la quantification de la densité microfilarienne (DMF) est également critique pour définir le traitement approprié. En effet, des effets indésirables graves peuvent survenir chez les individus porteurs d'une DMF élevée lorsqu'ils sont traités avec un médicament microfilaricide [3, 7, 16]. Ainsi, selon un protocole proposé en 2012, l'ivermectine n'est pas recommandée en cas de DMF supérieure à 30 000 Mfs par millilitre de sang (Mfs/mL), et la diéthylcarbamazine ne l'est pas si la DMF dépasse 2000 Mfs/mL [4]. De plus, si la DMF dépasse 8000 Mfs/mL, il est préférable de donner l'ivermectine en milieu hospitalier. Par ailleurs, même si la loase n'est toujours pas considérée comme une maladie tropicale négligée (MTN), elle constitue un obstacle majeur à l’élimination de l'onchocercose. En effet, l'extension des traitements de masse par ivermectine aux régions où celle-ci est hypoendémique n'est pas possible si la loase est également présente. La feuille de route 2021-2030 de l'OMS sur les MTN souligne que des diagnostics précis sont essentiels pour accélérer les progrès dans l’élimination des maladies et la réduction de la morbidité et de la mortalité [10].

Dans les régions endémiques, le diagnostic de l'infection à *L. loa* repose sur la recherche au microscope des Mfs présentes sur une GE calibrée préparée avec du sang périphérique prélevé entre 10 h et 16 h (pour tenir compte de la périodicité diurne de la microfilarémie à *L. loa).* Un petit volume de sang capillaire (généralement 50 microlitres, μL) est prélevé au bout du doigt à l'aide d'une lancette et d'un capillaire à hématocrite non hépariné, puis déposé et étalé sur une lame de microscope. Différentes méthodes existent pour l’étalement du sang (aucune n'est considérée comme la méthode « de référence »): la GE peut être circulaire, ovale, rectangulaire ou étalée en trois bandes parallèles. Dans tous les cas, la lame est laissée à sécher à température ambiante, déshémoglobinisée à l'eau, et colorée au Giemsa dans les 24 heures pour être finalement examinée en microscopie optique. Toutes les Mfs présentes sur la lame sont comptées. Le nombre est ensuite généralement multiplié par un facteur adéquat pour exprimer la DMF en Mfs/mL.

En France, pour que la technique quantitative d'un laboratoire médical soit accréditée, elle doit répondre à plusieurs exigences, notamment la précision, la fiabilité (répétabilité et reproductibilité), la robustesse et la corrélation avec une méthode déjà utilisée dans le laboratoire (le cas échéant) [23]. À partir de GE collectées en République du Congo lors d'un essai clinique, nous avons évalué la fiabilité de cette méthode diagnostique selon les critères recommandés par le Comité français d'accréditation (COFRAC) des laboratoires d'analyses médicales.

## Matériel Et Méthode

### Site, matériel et personnel de l’étude

Cette étude a été réalisée en avril 2021 dans le laboratoire du secteur opérationnel de Sibiti (3°41’06’’S, 13°21’04’’E), chef-lieu du département de la Lékoumou situé dans un environnement forestier. Les lames de GE utilisées ont été préparées lors d'un essai clinique évaluant la sécurité et l'efficacité du lévamisole chez des sujets infectés par *L. loa*. Cet essai, approuvé par le Comité d’éthique de la recherche en sciences de la santé (n° 226/MRSIT/IRSSA/CERRSSA), a reçu une autorisation administrative du Ministère de la santé et de la population du Congo (n° 469/MSP/CAB/UCPP-19). Lors de l'essai, 1876 GE contenant des Mfs de *L. loa* ont été collectées auprès des résidents de 21 villages situés dans un rayon de 40 kilomètres autour de Sibiti [6]. Deux techniciens congolais (PB et FL) ont effectué toutes les préparations et lectures des lames incluses dans les présentes analyses. Quarante lames ont été utilisées pour évaluer la fiabilité de la méthode et les 1876 lames ont été utilisées pour évaluer la variabilité inter-lecteurs.

### Examens parasitologiques

Cinquante µL de sang capillaire ont été prélevés par piqûre au bout du doigt et étalés sur une lame pour obtenir une GE de forme rectangulaire standardisée (3 cm par 2 cm). Les lames ont été séchées à température ambiante, déshémoglobinisées et colorées au Giemsa dans les 24 heures. Toutes les Mfs de *L. loa* ont été comptées à l'aide d'un microscope optique au grossissement x100. Toutes les DMF sont ici exprimées en Mfs/50 µL, et correspondent donc au nombre de Mfs comptées sur les lames.

### Accréditation des laboratoires en parasitologie

En France, l'accréditation d'une technique donnée par le COFRAC est une preuve de la compétence du laboratoire pour ladite technique. Elle délivre la certification de la norme internationale ISO 15189. Selon que la méthode d'analyse est qualitative ou quantitative, les démarches à effectuer pour obtenir la certification sont différentes. Dans le cas d'une méthode quantitative (comme dans la quantification microscopique des Mfs de *L. loa*), un certain nombre de mesures de performance sont demandées (lorsqu'elles sont applicables): spécificité de la méthode, fiabilité de la méthode, exactitude de la méthode, gamme analytique de la méthode, sensibilité (limites de détection et de quantification), linéarité de la méthode, contamination d’échantillon à échantillon, stabilité de la méthode, robustesse de la méthode, valeurs de référence, interférences éventuelles et corrélation avec une méthode de référence (ou une méthode déjà utilisée dans le laboratoire) [23]. Dans cette étude, nous avons évalué la fiabilité de la méthode (définie par la répétabilité et la reproductibilité), seul critère d'intérêt pour les méthodes de diagnostic microscopique.

### Répétabilité de la méthode

L’évaluation de la répétabilité consiste à réaliser plusieurs lectures d'un même échantillon dans des conditions d'analyse standardisées (même technicien, mêmes conditions matérielles et même jour de lecture). Habituellement, 10 mesures d'un même échantillon sont réalisées le même jour, par la même méthode; ceci étant fait sur au moins 2 échantillons, un de titre faible et un de titre fort. Dans le cas de la numération des Mfs de *L. loa*, le nombre de lames pouvant être lues dans une même journée est limité (entre 20 et 40 par jour en fonction de la DMF à mesurer) et dépend du lecteur (phénomène d'apprentissage du lecteur). Il n'est donc pas pertinent de faire relire une même lame 10 fois dans une même journée, c'est pourquoi notre test de répétabilité a porté sur 3 lectures successives d'un lot de 10 lames (et non pas seulement 2 lames lues 10 fois).

Dix lames anonymisées positives à *L. loa* ont été sélectionnées au hasard par un opérateur indépendant. Le test de répétabilité a été effectué 2 fois, c'est-à-dire avec 2 techniciens différents. Chaque technicien a lu la même série de 10 lames 3 fois le même jour, avec le même microscope. Après avoir été lues par un technicien donné, les 10 lames étaient restituées à l'opérateur indépendant, qui les mélangeait et ré-anonymisait avant de les redonner au même technicien pour une deuxième, puis une troisième lecture. Ces lectures répétées permettent d'obtenir des coefficients de corrélation de Spearman et des coefficients de variation (CV) de répétabilité, exprimés en pourcentages, qui sont calculés en divisant l’écart-type des mesures par leur moyenne (plus le coefficient est faible, meilleur est le niveau de répétabilité).

### Reproductibilité de la méthode

L’évaluation de la reproductibilité consiste à effectuer la lecture des mêmes échantillons dans trois conditions différentes: différents techniciens, différents matériels d'analyse et différentes dates de lecture. En microscopie optique, l’étude de l'influence du microscope n'est pas nécessaire si les microscopes disponibles sont considérés comme fonctionnels. Pour cette étude, l'analyse a été effectuée en utilisant 2 lots de 10 lames sélectionnées au hasard, toutes différentes de l'ensemble des 10 lames utilisées pour l’évaluation de la répétabilité afin d’éviter un éventuel biais d'apprentissage. Les deux techniciens ont lu le premier lot de 10 lames indépendamment, le même jour, en utilisant tous les deux le même microscope. Puis chacun des deux techniciens a lu le deuxième lot de 10 lames deux fois, en utilisant à chaque fois le même microscope mais à une semaine d'intervalle (les lames ont été ré-anonymisées entre les deux lectures). Chacun de ces deux tests (technicien différent et jour différent) permet d'obtenir un coefficient de corrélation de Spearman et un coefficient dit de fidélité intermédiaire, qui est obtenu en divisant l’écart-type des mesures par leur moyenne. La moyenne de ces deux coefficients donne le coefficient de reproductibilité (plus le coefficient est faible, plus le niveau de reproductibilité est élevé).

### Coefficients de variation acceptables

À notre connaissance, il n'existe pas de consensus sur les limites de variation acceptables pour la quantification de la DMF à *L. loa*, tant en ce qui concerne la répétabilité que la fidélité intermédiaire. En pratique, lorsqu'il n'existe pas de directive pour définir les coefficients acceptables, certains laboratoires de biologie médicale peuvent utiliser l’équation suivante:

$$f\left(x\right)\;=\;\frac{1}{\sqrt{x}}\;\times \;100$$

où x représente la valeur cible attendue (généralement, une valeur « calibrée » définie à l'avance par le laboratoire). Les variations du CV sont fonction du nombre d’éléments à compter: plus le nombre cible est faible, plus le CV attendu est élevé. Selon les recommandations de la Société française de biochimie clinique (SFBC), la limite d'acceptabilité de la fidélité intermédiaire ne doit pas être supérieure à 1,33 fois la limite de la répétabilité [25].

En parasitologie, la valeur cible attendue ne peut être définie à l'avance et il n'existe pas de lame « calibrée » avec un nombre défini de Mfs. Par conséquent, pour la répétabilité, nous avons décidé d'utiliser la formule suivante:

$$f\left(x\right)\;=\;\frac{\sum_{x_{\max}}^{x_{\min}}\frac{1}{\sqrt{x_{i}}}\times 100}{n}$$

où x_i_ est la moyenne des différentes lectures de chaque test et n est le nombre de lames lues. Pour la reproductibilité, nous avons utilisé l’équation suivante:.

$$f\left(x\right)\;=\;\frac{\sum_{x_{\max}}^{x_{\min}}\frac{1}{\sqrt{x_{i}}}\times 100}{n}\times 1,33$$

### Variabilité inter-lecteurs

Afin d'estimer la variabilité inter-lecteurs, nous avons réutilisé toutes les lames (N = 1876) collectées lors de l'essai clinique sur le lévamisole [6]. Au cours de cet essai, chaque lame a été lue par les deux techniciens le même jour, mais avec un microscope différent. Les proportions de lames où le nombre de Mfs dénombrées par chacun des deux techniciens engendrait un changement de protocole de traitement selon les seuils de DMF mentionnés dans l'introduction (2 000, 8 000 et 30 000 Mfs/mL) ont été calculées.

## Résultats

### Résultats de répétabilité

**Figure 1:**
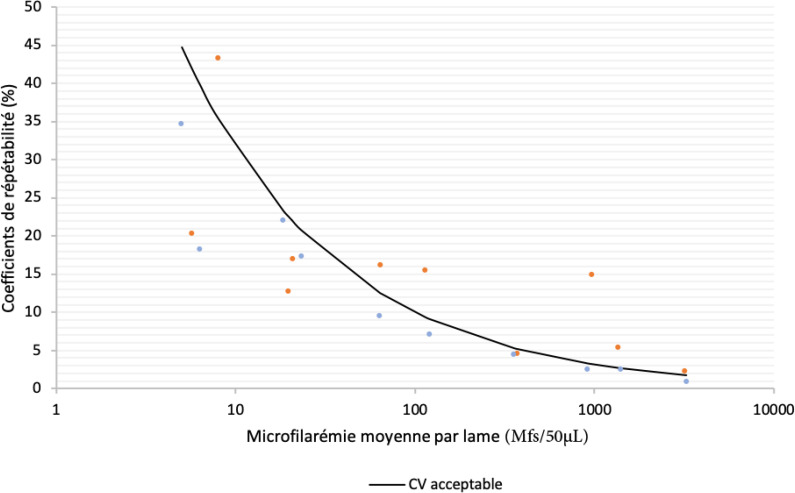
Comparaison des coefficients de répétabilité observés et acceptables. Les points orange représentent les lectures du technicien A, et les points bleus ceux du technicien B Comparison of observed and acceptable repeatability coefficients. The orange dots represent the readings of technician A, and the blue dots those of technician B

La médiane du nombre de Mfs comptées par le technicien A était de 88,7 Mfs/50 µL (de 5,7 à 3166,7 Mfs/50 µL). Les nombres ne sont pas tous entiers car ils sont calculés à partir des 3 lectures pour chaque lame. Concernant le technicien B, le nombre médian de Mfs comptées était de 92,2 Mfs/50 µL (de 5,0 à 3265,0 Mfs/50 µL). Les coefficients de corrélation de Spearman entre la première et la deuxième lecture, entre la première et la troisième lecture, et entre la deuxième et la troisième lecture étaient tous supérieurs à 0,975 (p < 0,0001) pour le technicien A. Pour le technicien B, ces coefficients étaient tous supérieurs à 0,999 (p < 0,0001). Les techniciens A et B présentaient des coefficients de répétabilité globaux de 15,3% et 11,9% (coefficient moyen = 13,6%) respectivement (Tableau I) alors que la limite acceptable du CV pour la répétabilité était estimée à 15,7% pour le technicien A et à 16,3% pour le technicien B (limite acceptable moyenne: 16,0%). La Figure 1 représente les coefficients de répétabilité observés et les coefficients considérés comme acceptables par rapport au nombre de Mfs à compter en appliquant la formule définie dans la section Méthodes.

$$f\left(x\right)=\frac{\sum_{x_{\max}}^{x_{\min}}\frac{1}{\sqrt{x_{i}}}\times 100}{n}$$

Toutes les lectures du technicien B ont des coefficients de répétabilité inférieurs à la limite acceptable définie (représentée par la ligne noire), ce qui indique une bonne répétabilité, alors que 6 des 10 lectures du technicien A ont un coefficient supérieur à la limite acceptable.

**Tableau I:** Résultats du test de répétabilité Results of the repeatability test

Technicien A				Technicien B			
Lame	Première lecture (Mfs/50μL)	Deuxième lecture (Mfs/50μL)	Troisième lecture (Mfs/50μL)	Lame	Première lecture (Mfs/50μL)	Deuxième lecture (Mfs/50μL)	Troisième lecture (Mfs/50μL)
1	5	7	5	1	6	6	3
2	10	10	4	2	7	7	5
3	22	20	17	3	19	22	14
4	17	21	24	4	27	24	19
5	70	70	52	5	70	63	58
6	124	123	93	6	127	124	111
7	389	371	355	7	373	355	341
8	1039	1065	801	8	934	919	890
9	1399	1380	1263	9	1449	1381	1400
10	3080	3212	3208	10	3275	3231	3289
Coefficient de répétabilité = 15,3%				Coefficient de répétabilité = 11,9%			
Coefficient de répétabilité global = 13,6%							

Pour le technicien A, le coefficient de répétabilité est calculé comme suit:
$$\mathrm{coefficient de répétabilité}\left(\%\right)=\frac{\sum_{i}^{1}{(}^{\mathrm{Moyenn}e_{i}}{/}_{\mathrm{Ecart}-\mathrm{typ}e_{i})}}{n_{i}}$$
où n est le nombre de lames (10) et i représente chaque lame entre 1 et 10. Mfs: microfilaires.

### Résultats de la reproductibilité

**Figure 2:**
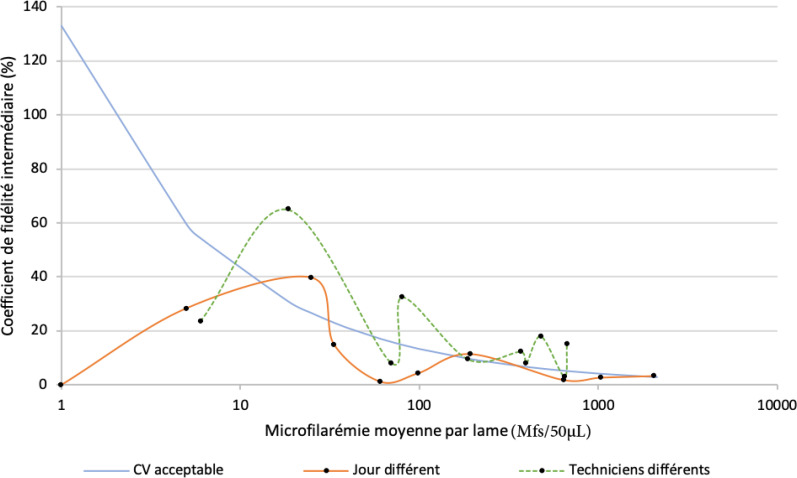
Comparaison des coefficients de fidélité intermédiaire observés et acceptables Comparison of observed and acceptable intermediate reliability coefficients

Le nombre médian de Mfs présentes sur les lames lues le même jour par deux techniciens différents était de 278,8 Mfs/50 µL (de 6,0 à 671,0 Mfs/50 µL). Le nombre médian de Mfs présentes sur les lames lues par un même technicien à une semaine d'intervalle était de 79,8 Mfs/50 µL (de 1,0 à 2048,5 Mfs/50 µL). Les coefficients de corrélation de Pearson entre les résultats obtenus le même jour par les deux techniciens et ceux obtenus à une semaine d'intervalle par un technicien donné étaient respectivement de 0,976 et 0,999 (p < 0,0001 pour les deux tests). Pour l’évaluation de la reproductibilité, lorsque la date de lecture ou le technicien était différent, les coefficients de fidélité intermédiaires étaient de 10,7% et 19,5% (coefficient moyen = 15,1%) respectivement (Tableau II). Les CV acceptables pour la fidélité intermédiaire ont été estimés à 29,4% pour différentes dates de lecture et à 15,6% pour différents techniciens (coefficient moyen = 22,5%). La Figure 2 représente les coefficients de fidélité intermédiaire observés et les coefficients jugés acceptables par rapport au nombre de Mfs à compter en appliquant la formule définie dans la section Méthodes:

$$f\left(x\right)=\frac{\sum_{x_{\max}}^{x_{\min}}\frac{1}{\sqrt{x_{i}}}\times 100}{n}\times 1,33$$

Lorsque le technicien ou le jour de la lecture change, l’écart par rapport à la limite acceptable semble ne pas être affecté par le niveau de DMF sur la GE.

**Tableau II:** Résultats du test de reproductibilité Results of the reproducibility test

Techniciens différents, même jour et même microscope		Jours différents, même technicien (A) et même microscope	
Technicien A (Mfs/50µL)	Technicien B (Mfs/50μL)	Premier jour (Mfs/50µL)	Deuxième jour (Mfs/50µL)
5	7	1	1
27	10	6	4
99	62	32	18
66	74	37	30
175	200	60	61
338	402	102	96
375	419	209	178
419	541	648	632
662	634	1050	1012
742	600	2095	2002
Coefficient de fidélité intermédiaire = 19,5%		Coefficient de fidélité intermédiaire = 10,7%	
Coefficient de reproductibilité = 15,1%			

Les coefficients de fidélité intermédiaire sont calculés comme suit:
$$\mathrm{Coefficient de fidélité intermédiaire}\left(\%\right)=\frac{\sum_{i}^{1}{(}^{\mathrm{Moyenn}e_{i}}{/}_{\mathrm{Ecart}-\mathrm{typ}e_{i})}}{n_{i}}$$
ou n est le nombre de lames (10) et i représente chaque lame entre 1 et 10.

### Variabilité inter-lecteurs

Le coefficient de corrélation de Pearson entre les deux lectures était de 0,961 (p < 0,0001). L'analyse de la variabilité inter-lecteurs de 1876 lames positives à *L. loa* lues par deux techniciens différents donne un CV moyen de 13,2%. Le CV global acceptable a été estimé à 18,6%. Parmi les 1876 CV estimés, 1219 (67,1%) étaient inférieurs à ce seuil de 18,6%, et la variabilité inter-lecteurs pouvait donc être considérée comme acceptable. Le Tableau III montre les CV observés et acceptables et les pourcentages de lectures avec un CV inférieur au CV acceptable en fonction du nombre de Mfs à lire sur chaque lame (les quatre catégories de DMF ont été définies selon les interquartiles). Lorsque l'on examine les résultats par catégorie de DMF, on observe que les lames à faible DMF étaient moins sujettes à une variabilité inter-lecteurs que les lames à forte DMF. Le pourcentage de lames pour lesquelles le CV calculé était inférieur au CV acceptable était très élevé, à 86,3% pour la catégorie des DMF les plus faibles et beaucoup plus faible (34,4%) pour la catégorie des DMF > 454 Mfs/mL.

**Tableau III:** Coefficients de fidélité intermédiaire en fonction des interquartiles des densités microfilariennes mesurées Intermediate reliability coefficients according to interquartiles of measured microfilaria densities

	Microfilarémie (Mfs/50µl)			
	1 – 34,5	35 – 139,5	140 – 454	> 454
CV acceptable moyen (%)	45,9%	16,0%	8,2%	4,4%
CV calculé (%)	20,9%	11,2%	9,2%	11,5%
Lame avec CV calculé < CV acceptable (N,%)	392 (86,3%)	365 (80,4%)	306 (67,4%)	156 (34,4%)

Comme mentionné plus haut, le protocole de traitement de la loase dépend de la DMF mesurée chez le patient [4]. Dès lors, le fait que les deux techniciens n'aient pas obtenu le même résultat pour une lame donnée aurait pu, pour certains sujets, avoir des implications en termes de traitement. Soixante (3,2%) individus avaient une discordance au niveau du seuil de 2000 Mfs/mL (100 Mfs/50 µL) dont 12 (0,6%) avec une discordance de DMF de plus de 50% entre les deux lecteurs; 59 (3,1%) individus avaient une discordance au niveau du seuil de 8000 Mfs/mL (400 Mfs/50 µL) dont 12 (0,6%) avec une discordance de plus de 50% entre les deux lecteurs et 32 (1,7%) avaient une discordance au niveau du seuil de 30 000 Mfs/mL (1500 Mfs/50 µL) dont 4 (0,2%) avec une discordance de plus de 50% entre les deux lecteurs.

## Discussion

À notre connaissance, ce travail constitue la première étude définissant certains des critères nécessaires à l'accréditation de la méthode de microscopie pour le diagnostic quantitatif de la microfilarémie à *L. loa.* Nous estimons le coefficient moyen de répétabilité à 13,6% et le coefficient moyen de reproductibilité à 15,1%.

Des études similaires ont déjà été réalisées pour évaluer la variabilité du diagnostic microscopique quantitatif du paludisme (densité des formes asexuées et sexuées de chaque espèce de paludisme observée sur une lame) [19, 20, 21]. Elles trouvent toutes des résultats similaires: (i) l'existence d'une variation inter-lecteurs substantielle dans le résultat quantitatif, (ii) une plus grande variabilité entre deux lecteurs qu'entre deux lectures par le même lecteur, révélant la nécessité à mieux former les microscopistes, et (iii) des variations plus importantes de résultats en cas de faible densité d'infection. Cependant, les études sur le paludisme ont utilisé le rapport des résultats obtenus au cours des lectures multiples comme indicateur de variabilité (un paramètre à caractère relatif, qui va donc surestimer les variations pour les petites densités parasitaires). Nous n'avons pas trouvé d’études utilisant la reproductibilité ou les coefficients de répétabilité comme estimateurs de la variabilité. De plus, contrairement au diagnostic de la loase, dans le diagnostic du paludisme, les lames ne sont pas entièrement lues et sont enregistrées comme négatives si aucun *Plasmodium* n'est vu dans 100 champs, ce qui peut augmenter la variabilité pour les petites charges (une chance accrue de ne rien trouver) par rapport aux charges élevées. Dans notre étude, les lames avec peu de Mfs étaient soumises à moins de variations que les lames avec un nombre élevé de Mfs, ce qui semble cohérent: plus il y a d’éléments à compter, plus il y a de chances d'en manquer, ce qui augmentera les CV.

Comme indiqué ci-dessus, il n'existe aucune référence permettant d’évaluer si nos CV sont acceptables ou non. À partir des méthodes utilisées par certains laboratoires, nous avons estimé les CV globalement acceptables à 16,0% et 22,5% pour la répétabilité et la reproductibilité, respectivement. En 2014, Ricos *et al.* ont réalisé une méta-analyse de la littérature scientifique sur différents paramètres de qualité évalués sur plus de 300 analyses biologiques [22]. La DMF à *L. loa* ne figurait pas parmi eux. Si l'on considère les méthodes de comptage par microscopie, les coefficients de répétabilité acceptables étaient de 28,0%, 21,0%, 10,2%, 17,8%, 9,1% et 17,1% pour les granulocytes basophiles, les granulocytes éosinophiles, les lymphocytes, les monocytes, les plaquettes et les granulocytes neutrophiles, respectivement. Enfin, dans le document COFRAC, SH GTA 04 [11], un exemple d'accréditation pour la lecture microscopique pour le diagnostic du paludisme indique un coefficient de répétabilité de 25,7%, jugé acceptable. Les CV acceptables que nous proposons pour la DMF à *L. loa* sont du même ordre de grandeur. Le COFRAC indique que des résultats similaires pour la répétabilité et la reproductibilité d'une méthode indiquent que celle-ci est robuste [11]. En effet, la répétabilité caractérise la meilleure performance possible (conditions optimales), tandis que la reproductibilité caractérise la performance analytique dans des conditions variables (opérateurs, équipement…). Dans notre étude, le coefficient moyen de répétabilité et le coefficient moyen de reproductibilité sont proches, ce qui indique que la technique est robuste (c'est-à-dire une méthode analytique dans laquelle les résultats obtenus sont jugés fiables même lorsqu'ils sont réalisés dans des conditions légèrement différentes). Ces bons coefficients de répétabilité et de reproductibilité sont importants, en particulier dans le contexte de la loase où la prise en charge individuelle des patients nécessite la connaissance de la DMF de l'individu, avec des stratégies thérapeutiques adaptées à des seuils de DMF prédéfinis pour prévenir les effets indésirables sévères post-ivermectine ou post-diéthylcarbamazine [4].

## Conclusion

Bien que l'absence de références de laboratoire ne nous permette pas de conclure sur la qualité globale de la quantification des charges de Mfs au cours de notre étude (menée dans une zone rurale de la République du Congo), nos données montrent des coefficients acceptables. Il est essentiel, surtout au vu des besoins croissants de diagnostic en Europe, de mettre en place un système de qualité et des procédures standardisées pour assurer un diagnostic quantitatif fiable aussi bien en Afrique centrale que dans les pays où la loase n'est pas endémique. Dans ce contexte, les outils de diagnostic biomoléculaire tels que la PCR en temps réel [14], la PCR nichée, les systèmes d'immunoprécipitation de la luciférase (LIPS) [5] ou plus récemment l'amplification isotherme médiée par les boucles (LAMP) [13] déjà utilisés pour le diagnostic du paludisme, pourraient également être envisagés pour des procédures de normalisation et d'accréditation supplémentaires.

## Remerciements

Les auteurs remercient l'ensemble du personnel du secteur opérationnel de la santé à Sibiti, République du Congo.

## Financement

Ce travail a été soutenu par l'Agence nationale de la recherche (ANR) (subvention numéro 18-CE17-0008).

## Contribution Des Auteurs

JTC a conçu et rédigé le protocole de l’étude.

FL, SB, FM et CBC ont révisé et validé le protocole de l’étude.

JTC, CBC, FM, FL et PB ont participé à la mise en place de l’étude.

FL et PB ont réalisé les lectures au microscope.

JTC a recueilli les données, réalisé les analyses et écrit le premier manuscrit.

JTC, FM, MB, SDSP, SB et CBC ont relu et corrigé le manuscrit.

Tous les auteurs ont validé le manuscrit.

## Liens D'intérêts

Les auteurs n'ont pas de conflits d'intérêts.
